# An Examination of the Experiences of Practitioners Delivering Sport Psychology Services within English Premier League Soccer Academies

**DOI:** 10.3390/sports10040060

**Published:** 2022-04-13

**Authors:** Francesca Dean, Emma Kavanagh, Amanda Wilding, Tim Rees

**Affiliations:** 1 Department of Rehabilitation and Sport Sciences, Bournemouth University, Bournemouth BH12 5BB, UK; awilding@bournemouth.ac.uk (A.W.); trees@bournemouth.ac.uk (T.R.); 2 Department of Sport and Event Management, Bournemouth University, Bournemouth BH12 5BB, UK; ekavanagh@bournemouth.ac.uk

**Keywords:** soccer, sport psychology, academy soccer, EPPP, psychological literacy, experiences of a sport psychologist, sport psychology integration

## Abstract

Sport psychology has become increasingly recognized and accepted within professional sports, including soccer. To date, there is a lack of research that examines the provision of sport psychology within elite soccer, particularly from the experience of applied practitioners working within the field. The current study adopted a qualitative, inductive approach, to examine the experiences of practitioners responsible for sport psychology delivery within elite soccer academies in England. Seven participants (four females; three males), working within academies in the English Premier League, took part in semi-structured interviews about their experience of delivering sport psychology services within elite soccer academies. Results demonstrated that the provision of sport psychology is continually evolving, yet there are a number of factors that appear to inhibit the full integration of the discipline into academy soccer. Six key themes were identified: The breadth of sport psychology provision; what is sport psychology; the stigma surrounding sport psychology services; psychological literacy; the elite youth soccer environment; and the delivery of sport psychology under the Elite Player Performance Plan. Participants identified a lack of psychological literacy among coaches and academy staff, as well as a low level of guidance regarding the provision of psychology within the England Football Association’s guiding document—the *Elite Player Performance Plan*—leading to considerable variation in the nature of the sport psychology provision. Future research would do well to also sample from a range of staff working within English soccer academies, in order to assess their perception of the level of provision and understanding of psychology.

## 1. Introduction

Although integration of psychological services within the preparation of soccer players has tended historically to lag behind a focus on physical, technical, and tactical aspects, sport psychology has become increasingly recognized and accepted within soccer [1,2] as it has in sport in general [3,4,5]. Indeed, although there is still some resistance towards employing sport psychology services [6,7], psychological skills are increasingly understood as fundamental for success and career progression in sports such as soccer [2,8,9]. As such, there is now an appreciation that successful performance is as much about a player’s overall psychological state and the development and execution of advanced psychological skills as it is about physical prowess. 

Since 2012, the English Premier League (EPL) has, via its Elite Player Performance Plan (EPPP), mandated the inclusion of psychology within the development program for English soccer players. Despite this initiative, the integration of sport psychology remains a challenge [2], and the discipline is not always positively received by all [10]. Additionally, there is limited research describing the experience of applied sport psychology practitioners working in the sport. The present study sought to gain insight into the delivery of sport psychology within the framework of the EPPP through capturing the experiences of sport psychology practitioners currently working within EPL soccer academies in the United Kingdom.

The EPPP was instigated in 2012, supported by a budget of GBP 320 million [11]. The main aim underpinning the development of the EPPP was to “increase the number and quality of [English] players gaining professional contracts and playing first team soccer at the highest level” [12]. This ambition would be realized through the development of athletes within the Premier League and Soccer League academies (the key development environment for young players in England), which are designed to provide young players with expert coaching, support, and education. Academies are audited every three years and must provide evidence of individualized coaching interventions that address each player’s physical, technical, tactical, and social and psychological development. Academies are subsequently awarded a categorization of 1 to 4 (1 being the highest), which then dictates the level of provision that is expected of them. As part of the EPPP guidelines, academies must make a sport science and medicine program available to all players. Such programs include physical testing and measurement, physiotherapy, medical services, nutrition, performance analysis, and psychology.

Although guidelines regarding the delivery of sport psychology within the EPPP were initially quite limited, the EPPP guidelines have more recently progressed, such that there are now more clearly defined minimum requirements. These include psychological testing, lifestyle management, and the delivery of mental skills education, as well as appropriate qualifications for the person delivering the psychological support. The latter means that those delivering sport psychology in English soccer academies must be qualified to a master’s degree-level in psychology or sport psychology, and registered on either the British Psychology Society or British Association of Sport and Exercise Sciences accreditation pathways.

Beyond the above minimum requirements, however, soccer club academies are afforded flexibility in delivery of psychological provision, such that there is no uniform content or standard of delivery. Some choose to draw on work focusing on the 5Cs of confidence, control, concentration, commitment, and communication [13,14], but otherwise evidence for the specific breakdown of psychology provision is scant [15]. The purpose of the 5Cs psychological intervention was to ensure that youth soccer players were provided with a greater amount of psychological education and development within training sessions [15]. Harwood et al. [15] suggested that their model was derived from Lerner et al.’s 6Cs of youth development—character, confidence, connection, competence, caring, and contribution [16]—developed with reference to the skills necessary to develop a more civilized society [15,16,17]. It is important to note, however, that it is difficult to assess the overall effectiveness of this model because there is no record of the number of clubs using it. Furthermore, the research examining the model is only being conducted by a small group of researchers [2,15,17,18].

More widely, coaches have long been considered a key source of support [19], who are often the “first line of defense” in dealing with psychological concerns. In this respect, there has been recent interest in the need for coaches and support staff in sporting organizations to develop their psychological literacy. The importance of psychological literacy, defined as “the ethical application of psychological skills and knowledge” [20] should not be underestimated. Although increasing the reach and impact of psychology through non-professionals (not just trained practitioners) would be a goal of developing psychological literacy, Murdoch [20] also noted the potential risks of non-professionals sharing psychological knowledge that may not be accurate. Indeed, Pain and Harwood [18] previously observed a lack of understanding and knowledge of sport psychology by coaches and support staff in English Soccer Academies, meaning they were potentially ill-equipped (even if they sensed the need) to draw on psychology within their coaching sessions. Thus, although the integration of psychological literacy across clubs is a positive steppingstone, it should not overtake the importance of having certified practitioners delivering psychological services to young players. The current research draws upon psychological literacy, that is, an individual’s ability to apply psychological understanding to their everyday (personal, social, and work) lives. The purpose of drawing upon psychological literacy is to provide relevant context to the study in relation to the understanding of psychology and how non-psychology practitioners then apply that knowledge.

The purpose of the present study was therefore twofold: first, to explore the role and experiences of practitioners working in sport psychology within elite soccer academies in England; and second, to understand the nature and implementation of sport psychology within the auspices of the EPPP. Furthermore, this study sought to explore these practitioners’ perceptions of what is successful and what needs further development in relation to the EPPP and how the academies are integrating sport psychology into their youth programs. 

## 2. Method

An interpretivist paradigm [21] was adopted to explore the experiences of practitioners responsible for sport psychology delivery within soccer academies in England.

### 2.1. Participants

Participants (four females, three males; aged 31 to 41 years, mean = 34.8) were British (all white), representing six elite English soccer academies at four Premier League (the highest-level league in England) and two Championship (the second tier of English football) clubs. Of the six academies, five had (at the time the research was conducted) Category 1 status (the highest-level academy in England, running on a scale from 1 to 3, as stipulated by the EPPP academy audit system); one had Category 2 status. All participants held a master’s degree or higher, three were British Association of Sport and Exercise Sciences (BASES) accredited, two participants were BASES Sport and Exercise Psychology Accreditation Route (SEPAR) accredited, and the same two were also Health and Care Professions Council (HCPC) registered. Participants’ experience of working as practitioners within a psychology unit ranged from 4 to 15 years.

### 2.2. Sampling

For inclusion in the study, participants were required to meet one key judgment criterion: participants needed to be responsible for delivering the sport psychology program to youth performers at an elite soccer academy with EPPP category status in England. In order to ensure that all those working within psychology departments at soccer academies had an equal chance to take part in the study, purposive chain sampling [22] was initiated during the data collection process. Participants recommended other practitioners who met the criterion of the study and who might be willing to take part. 

### 2.3. Procedure

Following ethical approval from an institutional research committee, an internet search using terms including “EPPP”, “soccer academies”, and “English soccer” was undertaken to identify all elite English soccer clubs with an academy holding EPPP status. Clubs which held the contact details for those working in the psychology department at each of the academies were listed, and clubs that did not display these contact details were removed. Potential participants were then contacted and invited to be interviewed for the study through an initial email. If a response was not received, follow-up contact was attempted, through email, two weeks later. Those who failed to respond were excluded from the study. Those who did respond and agree to be interviewed were sent a participant information pack, which contained details regarding the study aims, as well as information regarding anonymity and confidentiality. Participants were informed that they could withdraw from the study at any time, until the point of data analysis and write up. Prior to each interview, participants were provided with the opportunity to review the participant information sheet and give further verbal consent for participation.

### 2.4. Data Collection

Semi-structured interviews were undertaken to allow participants freedom to clearly explain their perspective and experiences, but the direction of the interview was still guided by pre-determined questions [21,23]. The interview guide was flexible, in order to allow each participant to share their own experience of providing psychological support, yet it centered around a number of key areas of interest. For example, the initial questions ascertained demographic information such as the participants’ role, their qualifications and years of experience within the field, and their experience of delivering sport psychology services to youth performers. Questions such as “Can you tell me about your career in sport psychology?” and “Can you tell me about the academy structure at your club and where you sit within it?” were used. The guide probed further in order to explore participants’ view of sport psychology and its current delivery in English Academy settings. It further examined the role of the EPPP in guiding such psychological provision. 

As each interview concluded, each participant was asked if there was anything that they wanted to add that had not previously been discussed; once answered, the interviewee thanked the participant and closed the interview. A number of probes, where appropriate, were used in order to elicit more depth from the participants, in order to gain a more detailed understanding of their perceptions and experiences. Interviews lasted between 60 and 90 min and were audio recorded. The interviews took place virtually, via video calls (rather than in person), due to restrictions on in person meetings due to COVID-19.

In order to ensure the trustworthiness of the data, the judgement criteria proposed by Smith and McGannon [24] were followed throughout the design and implementation of the study. It is anticipated that the sample will provide naturalistic generalizability across any other Category 1, 2, or 3 academy club that has EPPP status. However, due to the elite nature of the environment that those interviewed worked in, the study would not be transferable to grass roots or recreational soccer because the same dimensions and processes may not exist in those environments. Although qualitative research does not infer transferability per se, naturalistic generalizability of the results may be inferred through offering an insight into a variety of psychologists working across English elite soccer academies.

### 2.5. Data Analysis

In order to identify common themes within the data collected, in line with the interpretivist approach, thematic analysis was used as the framework of analysis [25]. Each interview was recorded and transcribed verbatim into a word document. Any irrelevant data (welcomes, inaudible audio, pleasantries) were removed in order to ensure the data analysis was meaningful and relevant to the study. As suggested by Braun and Clarke [26], a six-phase thematic analysis process was employed: (a) data familiarization and writing familiarization notes was achieved by listening to the interviews and transcribing them; (b) systematic data coding involved grouping common themes emerging from the raw data; (c) generating initial themes from coded and collated data was achieved through the codes being re-examined and patterns identified among the data; (d) developing and reviewing themes was completed when the identified themes were compared to the raw data, and looked over by the supervisory team, to ensure that they were appropriate; (e) refining, defining, and naming themes occurred once the themes were agreed upon; and, finally, (f) writing the report meant that all themes were presented and then discussed in detail. Although the process consists of six phases, each phase does not have to be isolated. According to Braun and Clarke [26], as the researcher becomes more familiar with thematic analysis, the six phases can become blended and carried out simultaneously. Following the interviews and transcription, in order to maintain anonymity, each participant was given a pseudonym. Using inductive thematic analysis, six key themes were identified. Within these key themes, raw data quotations were coded and grouped to form sub-themes.

## 3. Results and Discussion

From the interviews, 50 raw data codes were extracted and organized into six key themes: The breadth of sport psychology provision, what is sport psychology, the stigma surrounding sport psychology services, psychological literacy, the elite youth soccer environment, and the delivery of sport psychology under the Elite Player Performance Plan. Across these six themes, a further six sub-themes were generated which highlight additional contributing factors to two of the key themes (see Table 1).

### 3.1. The Breadth of Sport Psychology Provision

The initial theme which arose from participants was the breadth of sport psychology provision. This included three sub-themes: (a) the provision of sport psychology in academies, (b) the application of psychological skills, and (c) the barriers to the implementation of sport psychology. The current theme relates to the content that is being delivered to academy players and the way in which that is happening, as well as discussing the barriers that have the biggest effect on this. 

With regard to the provision of sport psychology across the six academies, it was apparent that the levels of provision varied greatly. When discussing differences between academy soccer and other youth sports, participants often discussed individual sports rather than other team sports. This is because there appeared to be more obvious differences in the provision of sport psychology in individual sports, and it was a common assumption that individual sports were significantly ahead of academy soccer in the provision of sport psychology.

#### 3.1.1. The Provision of Sport Psychology in Academies 

When discussing the provision of sport psychology, it is important to articulate the meaning of provision. For the purpose of the study, provision is referred to as the amount of sport psychology services that is provided to the players. This includes the form of delivery that is used, how much time is dedicated to delivering sport psychology, and the content that is covered within the sport psychology program. 

Samantha, who at the time of the study, had worked in the setting for eight years, explained:

9–11 [the age group], we do half and half, so half out on the pitch and half in the classroom. With the 12 s it’s again a mixture, so it’s half out on the pitch and half in the classroom. With the 13 s and 14 s at the moment it’s all classroom-based, just because of resources. 15 s, 16 s, again it’s a mixture, because we’ve got one member of staff who mainly focuses on those two, so it’s mainly in the classroom and out on the pitch. 18 s, their two and a half sessions a week is, err, classroom based.

Similarly, Tony, who has been at his current club for three seasons, described the way the sport psychology program is delivered to different age groups within an academy:

For the younger 9 s and 10 s, I’d run a monthly workshop, where I get them in weekly or bi-weekly. For the other age groups going up, where something I like to do is, and they have field focus as well. So, I don’t want psych to be in the class at all times, so I end up on the field. … So, I have, uh, about four periods where I’ll run through a couple of like challenges or a couple of skills and get all the players involved, with the main objective being a psychological one. Not looking at a textbook, although that’s working them as well, but they’re still working on their soccer. But the main objective would be that, working on communication, for example, or confidence when they want all the players to be the most confident player in the world, kind of thing. It seemed to buy into that a lot more than when it’s in a classroom. And then I say one-to-ones would be more for 18–23 s.

The way sport psychology is delivered appears to vary from academy to academy, and even from age group to age group. Dave explained that their sport psychology program had been designed with an over-arching framework in mind, thus evidencing more of a structural approach to delivery:

So, we have our kind of, I suppose, bespoke profile, if you want to call it that, that we use to identify key characteristics we believe are important to make it as a first team player within our club. So, we use that as our over-arching framework, and then from that we kind of filter it in, into learning plans, into targets, into goals, based on strengths and development areas as well.

Harry, who has been working as a practitioner in soccer academies since 2012, went further to explain that their sport psychology program had been designed to be a key part of the academy’s environment, taking a more holistic approach:

When it’s performance-related, and essentially, we do a series of things, we do workshops with players, with staff, we do individual support, we try and influence kind of the environment in some ways, so some of the projects we work on are more … kind of, systemic, so we try and influence the … I don’t want to go as far as to say the culture, that’s a bit extreme, but to kind of influence the staff to then influence the players, so we have an indirect impact, so we approach it in different ways, there’s lots of different strategies that, but yeah, some of the main things we use would be workshops, interactive workshops, player support, and yeah, kind of just creating, hopefully an environment whereby psychology is talked about and utilized as much as possible.

It was apparent from the interviews that the practice of psychology is diverse, encompassing team-based and individually tailored support, the delivery of psychological education, psychological skills training, and counselling services, alongside clinical referral or mental health support for players. 

#### 3.1.2. The Application of Psychological Skills 

Although sport psychology is often delivered through classroom-based workshops, an increasing number of academies are now focusing on integrating sport psychology during on-pitch sessions. Therefore, although the amount of official time allocated to sport psychology may lag behind other sport science disciplines, there are novel developments with regard to how sport psychology is being delivered. As Karen noted:

When we are out there, and we are talking, and we are doing things and then we might reflect afterwards and just try to attach anything to the actual game and making it as engaging and as real as physically possible.

Although there is an increasing focus on delivering sport psychology during on-pitch sessions, Dave explained the benefit of retaining classroom-based sessions. Dave stated that the academy players are educated regarding psychological skills and strategies, and then given the opportunity to practice them within a classroom setting before transferring them into training and game situations:

What we try to do is practice the techniques in the classroom. We might do different games, might do, err, just a drive through of the technique, then we’ll actually say ok when are these situations or what situations will arise where we might be able to use this technique? And then we go practice it for two or three weeks in training, and they can start to manipulate and practice it in games as well.

From these findings, it is evident that there is value in both classroom and practical sessions, especially when used concurrently; the classroom sessions provide a chance for information and techniques to be explained in detail, while the practical sessions allow for these techniques to be practiced within a controlled environment that can then be manipulated to reflect real match scenarios.

#### 3.1.3. Barriers to the Implementation of Sport Psychology

Barriers towards the use of sport psychology were reported by all participants, thus evidencing that these barriers are not just isolated to one club or category status, but instead are relevant across English soccer academies. Multiple barriers were reported including financial resources, staffing resources, and the relatively slow uptake of sport psychology. 

There was evidence in support of Pain and Harwood’s [18] assertion that a lack of financial resources was the most commonly perceived barrier to the implementation of sport psychology. As Dave explained:

So, I think probably limited resources would be the one area … I think a lot of it, in my, probably being controversial here, it’s down to funding a little bit, because if you do have the money you can buy better facilities, you can go abroad and potentially invite players—some clubs don’t have that luxury.

This was similarly noted by Harry:

I have experienced resources as a barrier. You know, you don’t need loads of money to implement psychology, I’m not saying that. But to do things in a certain way, having kind of funding backing, even if it’s to get things designed nicely or in a professional way.

As well as financial resources proving to be a barrier, it was reported that time and staff resources also had an impact on the delivery of sport psychology. Samantha stated, “With the 13 s and 14 s at the moment it’s all classroom-based, just because of resources. We haven’t got the time or the resources to be able to implement it out on the pitch all the time and with them.” In the same vein, Harry explained:

I think that the resources you have can sometimes dictate the delivery you can give. So, my previous experience was of me being alone as kind of being the single practitioner within that environment, so my time was spread a lot thinner, and so my strategy and approach had to be a lot different. I think with more staff and resources, you can do more with the players, because that’s when you can have a direct impact.

A number of participants reported that sport psychology still lagged behind other sport science disciplines in its level of integration into academy soccer development programs. As Lily, who at the time of the study had worked in the environment for seven years, stated, “Sport psychology is still late to the table, and we are still not there yet.” However, with the introduction of the EPPP, academies are now required to provide psychological support. Nonetheless, Lily did not credit the EPPP initiative for increasing such support, leaving questions regarding its impact upon the integration of sport psychology services into academy development programs. Samantha did, however, note a greater integration of sport psychology within soccer academies:

Probably over the last 18 months or so, maybe a little bit longer, psychology has been very much more integrated. So we are in the audit meetings, we are in the MDT [Multi-Disciplinary Team] meetings, we have our say, we are now working with the analysis team looking at the psychological behavior analysis and linking that up with the analysis team and clipping that footage so it can go into the prebrief and debrief. A lot of the things that coaches and the MDT are saying is psychological language … a lot of their CPD [Continuing Professional Development] incorporates psychology.

Samantha then added:

So, we are kind of riding the wave with that. I think we’re probably where sports science was five years ago, where S&C [Strength and Conditioning] was a bit of a taboo subject, but it’s on the horizon and it’s kind of on the up-and-coming, and I think psychology is the same at the minute. It’s still a bit of a taboo subject, but give it two or three years we’ll be up and coming, we’ll be forefront just like S&C.

Despite the latter quote concerning one specific club, it offers some evidence that the integration of sport psychology within academy soccer is progressing.

These results suggest that, in order for sport psychology to thrive within academy soccer, it still needs to take greater precedence, and to do so a lack of staffing and financial resources must be addressed. That is, clubs need to provide sufficient resources to deliver a successful program. That said, improving resources alone will not solve the issue of sport psychology integration—there also needs to be a greater level of acceptance from coaches and players in order to minimize the apparent resistance against sport psychology.

### 3.2. Understanding of Sport Psychology

According to Konter et al. [2], soccer psychology is multi-faceted, comprising social psychology, developmental psychology, clinical psychology, health psychology, and the psychology of coaching. Exposure to such a variety of psychological sub-disciplines may influence the way in which practitioners interpret the purpose and meaning of sport psychology. This theme’s key finding mirrors historical debates in the literature [27], in that, while being concerned with the psychological strategies and techniques to improve performance, sport psychology is also concerned with players’ overall wellbeing. As Lily stated, “For me, sport psychology, performance psychology, is like the interconnection of wellbeing and performance”. Similarly, Harry stated:

So, for me, sport psychology is about educating and supporting athletes and staff within a sporting environment. So, it’s about informing and upskilling them in psychology principles. It’s about, it’s about educating them in an appropriate way around what will impact their performance and what will impact the performance of the athletes they work with … and it’s about supporting appropriately so whether that’s supporting performance enhancement, and I also think it includes supporting wellbeing as well. So, I think both are really important, and I know different practitioners have different philosophies on that. For me they’re both completely linked, they’re both intertwined, performance and wellbeing. You can’t have one without the other. So, for me, both of those are really relevant to the role of a sport psych.

This quote thus underpins that, although sport psychology is of course concerned with athletes’ performance, its focus may also be taking a more holistic wellbeing approach [28,29]. Indeed, Sara noted that although sport psychology is concerned with enhancing performance, it is also about understanding athlete mental health:

Sport psychology is just looking at the mental aspect of their performance. … Sport psychology is not purely about how their mental side affects their sort of training and their performance on the pitch but also the wider aspects around the mental health, just like their daily wellbeing as well. It kinda (sic) just incorporates everything that they [the athletes] are. … The sport psychology will look more at the performance aspects, so looking at kind of like peak performance, optimal performance and those kinds of things.

The findings in this theme support the work of Moore and Bonagura [30], who stated that sport psychology is not solely for the purpose of performance enhancement, but instead seeks to support performers, their families, and their related organizations to optimize their level of functioning in a multitude of areas. Thus, while practitioners might think deeply about what psychology means to them [31], discussion then moved on to the level of psychological literacy among players and staff and the stigma that is still associated with the discipline. This demonstrates that, while sport psychology may be progressing in terms of awareness and acceptance, there is still a level of resistance from non-practitioners in relation to the implementation of sport psychology and the role of practitioners. 

### 3.3. The Stigma Surrounding Sport Psychology Services

Participants referred to a stigma associated with the use of sport psychology and the role of the practitioner that still persists in academy soccer. This supports the findings of Champ et al. [10], who stated that sport psychology is not always received positively by everyone. Lily noted experiences of such stigma: “They [Premier League personnel] said, well a sport psych is somebody that a player goes to for twenty minutes to be forced to see and then lies to them for twenty minutes and then leaves.” This negative attitude that players are “forced” to see a psychology practitioner and then “lie” suggests that players may still feel the need to avoid psychological support and would be reluctant to be honest when with the psychology practitioner. This showcases that players either believe that there is little value in speaking to a psychology expert, or they do not want the psychology practitioner to know the truth about how they are really feeling. Regardless, this demonstrates that a level of stigma persists around seeking psychological support. Such stigma was deemed to not just be present with academy players but also higher up in the professional soccer environment. Lily then explained:

I still think that the reason there aren’t as many sport psychologists is because it is still the unknown … so [coaches and players] will say we really value it, we really think it’s important, and we really buy into it, and we talk about it all the time and, you know. … However, we still are quite scared about what we do with it.

The above quote demonstrates that although coaches and players recognize the value of sport psychology, there remains a level of resistance to its adoption in soccer academies [6,7]. Harry reported that he still experiences a level of reluctance towards sport psychology: “I think psych is always that one that’s got a bit of, I guess stigma’s a word you could use for it, so there have [sic] been in my experience some reluctance.” Additionally, Samantha highlighted that players are still exposed to the stigma that seeking psychological support means that there is a problem, rather than the notion that the use of psychology can lead to performance gains:

So even though all the players know psychology is part of it [performance development] and all of that, you’ve still got that stigma behind that, go and see the psychologist because you’ve got an issue, and I think that’s still potentially what it is, rather than know that we are performance psychs (sic).

Participants noted that because players spend most of their time with their coach(es), these coaches have the potential to have more influence on the players than a sport psychologist. Sara stated, “It [the integration of sport psychology into training sessions] comes down to the coaches’ own philosophy.” Thus, if coaches do not consider sport psychology to be valuable, they may not include it. Karen provided insight as to why some coaches may be less likely to incorporate sport psychology into their training sessions: “Maybe sometimes you get the sort of coaches, who are typically in the professional development phase, so 18 s, the 23 s, seem to be ex-professionals, they are the ones that tend to be less open to change.” Sara noted: “Although you know what you should be doing, if the coach doesn’t then allow you to do it, it becomes like a little bit of conflict between what you know you should be doing versus what you’re actually doing”.

Our findings show how there still appears to be a stigma attached to the role of psychology and working with a sport psychologist. As Sara stated, “They still see it as they go to the shrink. … You go and see them when you’ve got a problem”. Additionally, it seems that the role of sport psychology is generally unclear to players. As Konter et al. [2] noted, sport psychology is not perceived as a regular performance aspect of development, but rather more often as a strategy for overcoming problems and helping the “problem athlete”.

### 3.4. Psychological Literacy

Psychological literacy was noted by the participants as the level of understanding a person has of sport psychology and their ability to apply that understanding. In this case, discussing the current level of psychological literacy participants believed coaches and players had, Lily stated, “Actually, the awareness and psychological awareness and literacy, I don’t think it’s, I don’t think it’s where it could be. I think it could be improved, hundred percent, on what it is”. Although Sara suggested that coaches’ psychological literacy is poor, she had a different stance on where the problem lies: “So, their [coaches’] awareness of it [sport psychology] is high, their understanding of it is low … but if you ask them, they would say they have a very high understanding of it. So, I don’t think they realize quite how complex it is”. As this quote demonstrates, it would appear that coaches have a high level of awareness of sport psychology, in that they are exposed to sport psychology within academy soccer. However, as Sara stated, these same coaches may also mistakenly believe they have a high understanding of sport psychology. This highlights the value of coach education and workshops and the need to educate coaches about the fundamentals of sport psychology, how to use it, and the benefits that surround it [32]. This in turn could provide coaches with a greater understanding of sport psychology and the tools to successfully incorporate it into their coaching sessions. 

In a similar vein, Karen explained a lack of psychological literacy in simpler terms: “I think there will be some staff where it’s quite new and the topics are not gonna (sic) be particularly familiar … they might have heard of the terms but not know what they mean and what it looks like in practice”. This is an important consideration that is supported by Pain and Harwood [18], who stated that those coaches with a low knowledge base and understanding of sport psychology are ill-equipped to deliver such skills within their training sessions. As recognized by Murdoch [20], this has the potential to be problematic, because inappropriate (and incorrect) information and skills can be passed onto the athletes, which can even be detrimental for their performance and wellbeing. The present study’s findings demonstrate an awareness of sport psychology but also a lack of understanding among coaches in relation to the application of sport psychology within their coaching practice. As will be discussed in the later sub-theme “Freedom in delivery”, coach and player understanding of sport psychology may be affected by the varied psychological programs employed across club academies, meaning there is no standardization in exposure of coaches and players to sport psychology.

### 3.5. The Elite Youth Soccer Environment

As it is in sport in general [33], elite youth soccer is characterized by a highly pressurized climate for success [34]. This characteristic remains true of the English soccer academies highlighted in the current research, with a primary focus on results, potentially at the expense of player development.

Participants noted that although the development of players is important, the focus of academy soccer remains on results and winning. This is evidenced by Sara:

I think the reality of it is that it’s still very competitively orientated. It’s still very ego oriented, it’s still very much a win culture. Although they are looking to develop the players, um, it’s not purely developmental orientated. Whilst they are developing the players, I think the winning aspect is still the primary focus.

Although Sara stated that there is consideration of player development within the academies, the fact that the primary focus is on results may be potentially problematic, because this may mean that other factors, such as player wellbeing, may be neglected. In a similar vein to Sara, Lily stated that the level of competitiveness is related to the idea of academies operating as businesses.

It’s much more of a business than I gave it credit for when I started, there’s a massive element of, you know, is it gonna (sic) make us money, is it gonna (sic), is he gonna (sic) go to our first team, are we gonna (sic) be able to sell him for a profit, much more so than I probably wanted to believe when I started.

This statement echoes previous work noting that soccer clubs maintain a primary focus on achieving the best possible results and winning each game [35].

In a similar vein, Harry stated the importance of understanding that some staff and players may find the academy soccer environment significantly challenging:

It can be a really difficult environment as well. I think there’s, it’s, it would be remiss to ignore that some people really struggle with this environment. … There’s a lot of pressure, whether it’s from themselves [academy players], whether it’s from their parents, whether it’s from other people involved in their journey, to kind of be a certain way, to perform a certain way, and for some players, that’s not for everyone. … It’s a high expectation environment that’s not for everyone, and again some staff thrive on it, some staff don’t. So I think for both it’s one of those that can go either way.

This notion is further supported by Tony, who explained the difficulty that staff working within the environment encounter:

The environment, soccer generally, as in the organization, is a very cut-throat environment. It’s yeah, it’s not too forgiving. But at the same time, within your organization everyone’s quite close. Like when you have a lot of, yeah, relationships between people can be really good. But then at the same time, if those people don’t do well, then potentially you could lose your job. You know. Which might have no relation to you. So that’s part of the environment. I think it can be quite hard I think for the coaches as well, all staff, not just coaches.

It is evident that the academy soccer environment is challenging, highly competitive, and places considerable demands on both athletes and support staff [36].

### 3.6. Delivery of Sport Psychology under the EPPP

When conducting research around sport psychology within English soccer academies, it is important to discuss the presence of the EPPP, because, as the overarching framework for player development in English academies, it mandates the minimum requirements for psychology provision. When discussing the EPPP and the delivery of sport psychology within soccer academies, three sub-themes emerged: (a) lack of guidance; (b) freedom in delivery; and (c) room for development.

#### 3.6.1. Lack of Guidance

Participants expressed their sense of the inadequacy of the EPPP as a guidance framework for standardized provision of sport psychology within soccer academies. Many of the participants shared concerns surrounding the EPPP as a guiding document, given its lack of (age appropriate) guidelines. As Champ et al. [36] purported, to date the EPPP has not yet achieved what it originally set out to in terms of guiding psychological practice in academies. This is reflected in the following extract from Sara:

I guess, it’s [the psychology section of the EPPP] vastly inadequate, in terms of when we get audited and I look at the one-page Premier League document, whereby it says, have you seen it, you’ve seen it right? ‘phychology’ (sic) … do I need to say any more?

This was supported by Samantha, “Within the audit process, they spelt psychology wrong, so I mean they obviously don’t have a great understanding. They want to include it, but I don’t think they have a full understanding of it”. Lily then continued to explain why she believed the psychology section of the EPPP to be inadequate:

It was written by a sport scientist, so the language and everything like that, in terms of testing is very physically driven, and all they’ve done is they’ve picked up the sport science kind of language, if you like, and they’ve dumped it onto a page that’s ‘psychology’, and they can’t even spell psychology right, so let’s not go there.

The above quote suggests a lack of expertise in the creation of the psychology section of the EPPP. All of our participants suggested that the EPPP currently fails to include sufficient detail to scaffold the delivery of standardized provision of sport psychology across academy settings.

#### 3.6.2. Freedom in Delivery

Alongside criticisms of the EPPP as a guiding document, some participants reframed this more positively, because it gave them freedom in their delivery of sport psychology. As Karen noted:

I think that the psychology section of it [EPPP] is one of the most vague … and I think that might frustrate people, but I think it might also be a good thing. … I think the pure performance psychology section is sort of one page, so it gives us a little bit of a framework and the minimal thing we’ve got to do, but how we do it is entirely up to us.

Lily similarly stated, “There was a lot of freedom given to clubs on how they wanted to do it, so that is based on the philosophy of the club and resources, of course, and funding and where they want to do things”. The freedom noted by Karen and Lily might be problematic, if all clubs deliver psychology differently, without any form of standardization. However, Karen further stated that such an approach may be preferable than a stricter set of guidelines: “I think if it [EPPP] had a lot more detail and was a lot more prescript (sic), I think you’d lose that autonomy as a practitioner, and I think the role would be a lot less enjoyable because you’d feel like you were ticking boxes all the time”.

Although it was noted that the EPPP lacked detail and specificity, in some cases, this was viewed as beneficial, because this gave psychology practitioners greater flexibility and ownership over their delivery of sport psychology, allowing them to better cater to the needs of the academy and players. In some ways this mirrors the concept noted by others (e.g., see Daley et al. [37]), that although psychological models and frameworks can be useful as a starting point for applied practice, practitioners should indeed tailor such content to the context in which they are working. That said, this lack of detail and specificity may also lead to a lack of standardization across academies in terms of the psychological content and support that players receive, and thus our results suggested there was still room for development regarding the EPPP and its sport psychology guidance.

#### 3.6.3. Room for Development

Participants noted that implementing the EPPP guidelines could become a “tick box exercise”. As Lily stated, “The first thing that always springs to my mind [in relation to the EPPP], rightly or wrongly, is tick box”. Samantha noted that this can lead to academies not going further in their provision of psychology in their programs: “Clubs are then using it [EPPP] as a tick box exercise, so they never fully integrate it themselves”. Dave did not feel constrained by the guidelines, however, stating:

Well, it is embedded, but I wouldn’t go in to a one-to-one with a player or a workshop thinking, ‘oh is this going to be ok for EPPP?’ Uh, I think I kind of have faith in the program that we have meets EPPP requirements, rather than trying to make it fit in to the EPPP.

Conversely, Sara stated, “So, I would say it’s given us a little bit more structure, but in terms of what we deliver and how we deliver it, it has no impact whatsoever”.

Overall then, these findings suggest that the guidance for sport psychology within the EPPP is considered somewhat sub-optimal, but that the lack of detail and specificity may not necessarily be a negative. Indeed, with this lack of detail and specificity, sport psychology practitioners may have more freedom to design their psychology program to best meet the needs of their academy. However, even with the above said, according to this study’s participants, at least, further development of the EPPP guidance appears warranted, in particular with greater input from experts in psychology.

## 4. Conclusions

In addressing the purpose of our study, we sought to examine the experiences of sport psychology practitioners working within elite soccer academies in England. Across the six themes presented, our findings highlight the rich and diverse nature of psychological service delivery within these settings. Psychological practice encompassed team-based and individually tailored support, the delivery of psychological education, psychological skills training, and counselling services, alongside clinical referral or mental health support for players. Practitioners highlighted their responsibility for both performance enhancement and player welfare—a critical shift in service delivery. Ecological frames of reference were adopted, whereby participants acknowledged that while they worked with athletes this happened within academy environments with cultures and norms that have an impact on their practice and the behavior(s) of the athletes with whom they work. As such, participants recognized how, in modern settings, psychology practitioners are responsible for supporting wider cultural development in clubs and academies, alongside education of support staff and coaches. It is evident that practitioners working in soccer academy settings do so in dynamic environments—psychological practice is varied and ever evolving, and there remains no single way of being a psychologist in this setting [38].

The results demonstrated that, although the provision of sport psychology is improving and continuing to develop, there remain a number of factors that inhibit a full integration of the discipline into the academy development program. The most prominent factors identified in this study were the level of psychological literacy among coaches, and the attention and resources dedicated to the discipline. That is, in order for the integration of sport psychology into the wider development program to be successful, those who are responsible for aiding the promotion and delivery of psychological content require a greater level of understanding than is currently present. In line with the findings of Murdoch [20], our study found that the level of psychological understanding among coaches appeared to be lower than the coaches perceived it to be. This is potentially problematic due to the level of impact and influence that coaches have on players [39], and the fact that they are such a key source of support [19]. If, due to a lack of psychological literacy, coaches are promoting sub-optimal psychological advice, then players may be at risk of developing poor psychological habits. Thus, in order for sport psychology to be successfully and safely provided to academy soccer players, practitioners should be employed to deliver such content, but further should have a significant role in the education of coaches and other support staff about sport psychology and the importance of delivering sound psychological advice [40]. Additionally, from the findings, it is evident that there is still a stigma attached to the discipline of sport psychology. This stigmatized thinking and the fear of the unknown is still causing a level of resistance from some coaches and players when seeking psychological support [41]. Therefore, a recommendation of this study would be to provide education to players and coaches around sport psychology and the way in which it can benefit performance, in order to help combat such thinking.

As well as examining the general experiences of sport psychology practitioners, we also sought to explore participant experiences of working under the auspices of the EPPP—the English Premier League’s guiding document concerning player development—of which psychology is a core component of sport science support. Theme six specifically reflected this experience. Although participants acknowledged that, since its inception, the guidance for sport psychology delivery had been improved, they felt that it still lagged behind other sport science disciplines in depth and/or quality of the syllabus, thereby limiting its potential to guide the delivery of psychological services. A key suggestion echoed by all participants was that in order to improve the integration of sport psychology services within academy soccer, the psychological guidance within the EPPP would benefit from being significantly revised. The evidence from the present study suggests that the EPPP documentation, in its current format, provides inadequate guidance in relation to the content that should be delivered and how it should be delivered. Such revision of the EPPP guidelines would help to extend the current minimum standards of psychological provision and would also create a level of uniformity across academies within each category, thereby increasing the focus on developing psychological literacy in players. Along these lines, participants noted their preference for the EPPP guidance for sport psychology to be revised and re-written by leading practitioners who are familiar with the discipline and who have experience of working within academy settings. This would enable the adoption of correct terminology as well as providing clear guidance with a contextual understanding of the demands of such settings. By way of example, further guidance with regard to employing a holistic, player-centered approach, which focuses on well-being as well as performance, would be important. In addition, the provision of one-to-one support alongside more general educational sessions, and on pitch-based sessions, with monitoring of player psychological development, would be important [36,42]. Increasing guidance with regard to the emotional and cognitive maturation of players, alongside an understanding of adolescent psychological development, would provide the opportunity for psychological service delivery that meets the changing demands of youth performers across critical stages of their (player) lifespan. The EPPP, with revision, thus has potential to encourage service provision that genuinely supports youth players within and beyond soccer environments.

The results of this study could aid in the future refinement of the psychological elements of the EPPP. Indeed, a strength of this study is that data were collected from seven practitioners, working across a number of different academies, with representation of both male and female participants. Thus, the results from this study should transfer to any academy with EPPP Category 1 or 2 status, or indeed to Category 3 academies attempting to reach Category 2 status. With the above said, a limitation is that a number of practitioners whom we approached either (a) did not respond to our invitation or (b) did respond but did not participate. Thus, our sample was to an extent self-selected, such that our results may simply reflect those more willing to speak out about their experiences [43]. Overall, this study has helped to deepen our understanding of the role of practitioners responsible for the delivery of sport psychology within English academy soccer. Future research would do well to also examine perceptions from a range of staff working within English soccer academies, in order to assess their perception of the level of provision and understanding of psychology.

## Figures and Tables

**Table 1:** Themes and sub-themes described by the sport psychology practitioners.

Theme	Sub-Theme	Description
The breadth of sport psychology provision	The provision of sport psychology in academies	Psychological practice is diverse, encompassing team-based and individually-tailored support, the delivery of psychological education, psychological skills training, and counselling services, alongside clinical referral or mental health support for players.
	The application of psychological skills	There is value in both classroom and practical sessions, especially when used concurrently. Classroom sessions provide theory, while practical sessions allow for the development of skills in a controlled setting.
	Barriers to the implementation of sport psychology	Clubs need to provide sufficient resources to deliver a successful program. At present, there is an apparent lack of staffing and financial resources dedicated to sport psychology.
Understanding of sport psychology		Although sport psychology may be progressing in terms of awareness and acceptance, there is still a level of resistance to the implementation of sport psychology and the role of practitioners, linked to a narrow understanding of the breadth and scope of psychological support.
The stigma surrounding sport psychology services		There still appears to be a stigma attached to the role of psychology and sport psychologists. The role of the psychologist is still unclear to players and is often seen as a way to solve problems, rather than as a holistic aspect of player development and performance programs.
Psychological literacy		There is an awareness of sport psychology, but this remains narrow. Psychological literacy is currently lacking in academy settings, especially within core support staff, such as coaches.
The elite youth soccer environment		Academy cultures have an impact upon the delivery and reception of psychological support. It is evident that the academy soccer environment is challenging, highly competitive, and places great demands on both athletes and support staff.
Delivery of sport psychology under the EPPP	Lack of guidance	It was commonly reported that the EPPP fails to include sufficient detail to scaffold delivery of standardized provision of sport psychology within soccer academies.
	Freedom in delivery	In some cases, the lack of detail and specificity of the EPPP was viewed as beneficial, because it gives psychology practitioners greater flexibility and ownership over their delivery of sport psychology, allowing them to better cater to the needs of the academy and players.
	Room for development	Further development of the EPPP guidance is required, with greater input from experts in the field, in order to ensure the guidance is relevant and detailed.

## Data Availability

The authors confirm that the data supporting the findings of this study are available within the article.
